# Systemic Fibrinolysis in Symptomatic Intermediate-Risk Pulmonary Embolism: A Real-World Cohort Study

**DOI:** 10.3390/jcm15082932

**Published:** 2026-04-12

**Authors:** Eva Cervilla-Muñoz, Pablo Demelo-Rodríguez, Rubén Alonso-Beato, Miriam Juárez-Fernández, Iago Sousa-Casasnovas, Lucía Ordieres-Ortega, Marina López-Rubio, Luis-Antonio Alvarez-Sala Walther, Francisco Galeano-Valle

**Affiliations:** 1 Venous Thromboembolism Unit, Internal Medicine Department, Hospital General Universitario Gregorio Marañón, 28007 Madrid, Spain; eva.cervilla@salud.madrid.org (E.C.-M.); ralonsob@salud.madrid.org (R.A.-B.); lucia.ordieres@salud.madrid.org (L.O.-O.); marinalopezrubio@outlook.com (M.L.-R.); luisantonio.alvarezsala@salud.madrid.org (L.-A.A.-S.W.); francisco.galeano@salud.madrid.org (F.G.-V.); 2 School of Medicine, Universidad Complutense de Madrid, 28040 Madrid, Spain; miriam.juarez@salud.madrid.org (M.J.-F.); iagocasasnovas.sousa@salud.madrid.org (I.S.-C.); 3 Instituto de Investigación Sanitaria Gregorio Marañón, 28007 Madrid, Spain; 4 Department of Cardiology, Hospital General Universitario Gregorio Marañón, 28007 Madrid, Spain; 5 Centro de Investigación Biomédica en Red de Enfermedades Cardiovasculares (CIBERCV), Instituto de Salud Carlos III, 28029 Madrid, Spain

**Keywords:** anticoagulation, mortality, systemic fibrinolysis, pulmonary embolism

## Abstract

**Background**: The role of systemic fibrinolysis in patients with intermediate-risk pulmonary embolism (PE) remains controversial because of the uncertain balance between potential benefits and bleeding risk. This study evaluated the association between systemic fibrinolysis and clinical outcomes in a real-world cohort of patients with symptomatic intermediate-risk PE. **Methods**: This prospective observational study included consecutive patients with symptomatic intermediate-risk PE from 2009 to 2019 at a tertiary hospital. Patients receiving systemic fibrinolysis were compared with those treated with anticoagulation alone. The primary outcome was 30-day all-cause mortality. Secondary outcomes included major bleeding, and recurrent venous thromboembolism. Multivariable Cox proportional hazards regression models were used to adjust for potential confounders. **Results**: A total of 560 patients with symptomatic intermediate-risk PE were included, of whom 54 (9.6%) received systemic fibrinolysis. Patients receiving fibrinolysis were younger than those treated with anticoagulation alone (median age 58 vs. 73 years; *p* < 0.001). Thirty-day mortality occurred in 1.8% and 3.3% of patients, respectively (*p* = 1). After adjustment, fibrinolysis was not associated with reduced 30-day mortality (aHR 1.5; 95% CI 0.1–17.9), nor with a significant increase in 30-day major bleeding (aHR 2.6; 95% CI 0.8–8.3). Intracranial hemorrhage and VTE recurrences were rare. **Conclusions**: In this real-world cohort, systemic fibrinolysis was not associated with improved survival compared with anticoagulation alone, consistent with current guideline recommendations against routine fibrinolysis in intermediate-risk PE.

## 1. Introduction

Venous thromboembolism (VTE), which comprises pulmonary embolism (PE) and deep vein thrombosis (DVT), remains a major contributor to cardiovascular morbidity and mortality worldwide [1]. Among its clinical manifestations, PE is associated with the highest short-term mortality and represents a medical emergency requiring prompt evaluation and management [2]. Given the wide spectrum of PE, early and accurate risk stratification is essential to guide therapeutic decision-making [3].

Current guidelines stratify patients with acute PE into high-, intermediate-, and low-risk categories based on hemodynamic status and markers of right ventricular (RV) dysfunction or myocardial injury [3]. Patients presenting with hemodynamic instability are classified as high-risk and require urgent reperfusion therapy, most commonly systemic fibrinolysis. In contrast, the majority of patients are hemodynamically stable and fall into the intermediate- or low-risk groups [4].

Patients with intermediate-risk PE are characterized by preserved systemic blood pressure but evidence of RV dysfunction and/or myocardial injury. Although initially stable, this subgroup carries a non-negligible risk of early clinical deterioration compared with low-risk patients [5,6,7,8,9]. This has prompted an interest in systemic fibrinolysis as a potential strategy to reduce thrombotic burden, improve pulmonary perfusion and prevent hemodynamic decompensation [10]. However, fibrinolytic therapy may also increase the risk of major bleeding, including intracranial hemorrhage [11].

Evidence from randomized clinical trials evaluating systemic fibrinolysis in intermediate-risk PE has been inconsistent. While some studies have shown a reduction in hemodynamic decompensation, this has not been consistently accompanied by a survival benefit and has been associated with an increased risk of bleeding complications [10,11,12,13,14,15]. The PEITHO trial [10], in particular, demonstrated a reduction in clinical deterioration at the expense of a higher incidence of major bleeding and intracranial hemorrhage. As a result, current guidelines recommend anticoagulation as the standard therapy for most patients with intermediate-risk PE and reserve reperfusion therapies for those who develop clinical worsening despite anticoagulation [3,16].

In recent years, research has increasingly focused on alternative reperfusion strategies, including catheter-directed therapies and reduced-dose fibrinolysis, with the aim of improving the balance between efficacy and safety in this patient population [17,18,19,20].

Despite the availability of randomized evidence, the management of intermediate-risk PE in routine clinical practice remains heterogeneous. Patients enrolled in clinical trials are often highly selected, which may limit the applicability of their findings to unselected populations encountered in everyday practice. In this context, observational studies can provide complementary insights by reflecting real-world treatment decisions, patient characteristics, and outcomes.

Therefore, the aim of this study was to evaluate the association between systemic fibrinolysis and clinical outcomes in patients with symptomatic intermediate-risk PE in a real-world clinical setting.

## 2. Methods

### 2.1. Study Design

This was a single-center, prospective observational cohort study including consecutive patients with acute intermediate-risk PE diagnosed between 1 January 2009 and 31 December 2019, at a tertiary hospital in Spain.

### 2.2. Patients and Follow-Up

Adult patients with objectively confirmed acute symptomatic PE were eligible for inclusion. PE was diagnosed by computed tomography pulmonary angiography (CTPA) showing intraluminal filling defects in the pulmonary arterial tree.

Patients were classified according to the 2019 European Society of Cardiology (ESC) risk stratification criteria [3]. Only patients with intermediate-risk PE, defined as hemodynamically stable PE with a simplified Pulmonary Embolism Severity Index (sPESI) score > 0 and/or evidence of RV dysfunction and/or myocardial injury, were included. RV dysfunction was defined according to ESC criteria as the presence of at least one of the following: an RV-to-left ventricular (LV) diameter ratio ≥ 1.0 on computed tomography pulmonary angiography or echocardiographic evidence of RV impairment [3]. Myocardial injury was defined by elevated troponin I or high-sensitivity troponin I.

The Shock Index was calculated as the ratio of heart rate to systolic blood pressure; values greater than 0.9 were considered elevated [21].

Patients with incidental or asymptomatic PE diagnosis and patients who were hemodynamically unstable at presentation were excluded. Hemodynamic instability was defined as systolic blood pressure < 90 mmHg for more than 15 min not attributable to hypovolemia, sepsis, or arrhythmia.

All patients were prospectively enrolled through an institutional VTE registry that captures patients managed by the hospital’s VTE unit, including both hospitalized patients and those discharged from the Emergency Department and subsequently evaluated in the outpatient VTE clinic. Consecutive enrollment was ensured through a standardized institutional care pathway by which all imaging-confirmed PE cases were automatically referred to the VTE unit, regardless of care setting or time of presentation.

Patients were followed for at least 30 days after PE diagnosis.

### 2.3. Data Collection

Clinical, laboratory, imaging, treatment, and outcome data were prospectively recorded in a dedicated database. Collected variables included demographic characteristics, comorbidities, clinical presentation, VTE risk factors, imaging findings, treatment strategies, and 30-day clinical outcomes.

PE location was classified according to the criteria proposed by Jain et al. [22] as central when emboli involved the pulmonary trunk and/or main pulmonary arteries, or peripheral when emboli were located in the lobar, segmental, or subsegmental arteries.

### 2.4. Treatment

All patients received anticoagulation therapy according to standard clinical practice. Systemic fibrinolysis was administered either at initial presentation or during early hospitalization, at the discretion of the treating physician. Clinical management followed institutional protocols, although treatment decisions were ultimately individualized based on physician judgment and patient characteristics.

In most cases, fibrinolysis was initiated shortly after diagnosis in patients with markers of higher severity within the intermediate-risk category. In some patients, fibrinolysis was administered later due to clinical deterioration, including hemodynamic worsening, increasing oxygen requirements, or progression of RV dysfunction.

In general, the decision to administer fibrinolysis was based on the presence of markers of higher severity, including RV dysfunction on echocardiography or CT pulmonary angiography, elevated cardiac biomarkers, higher heart rate, lower systolic blood pressure, central emboli location, and overall clinical assessment of hemodynamic compromise (including an elevated shock index). No standardized protocol defined the timing or specific criteria for fibrinolysis administration.

According to the institutional protocol, the fibrinolytic agent used was recombinant tissue plasminogen activator (rt-PA; alteplase), administered at the recommended dose of 100 mg intravenously over 2 h.

### 2.5. Outcomes

The primary outcome was 30-day all-cause mortality. Secondary outcomes were: (1) early major bleeding; (2) identification of risk factors for early bleeding; and (3) early recurrent VTE.

Recurrent PE was defined as a new filling defect on CTPA or a new ventilation/perfusion mismatch on lung scintigraphy. Recurrent DVT was defined as thrombosis in a new venous segment compared with the initial event, confirmed by duplex ultrasonography.

Major bleeding was in accordance with the International Society on Thrombosis and Haemostasis definition as clinically overt bleeding associated with a hemoglobin decrease ≥ 2 g/dL, transfusion of ≥2 units of red blood cells, involving of a critical site, need for surgical intervention, or fatal outcome [23].

Data on PE diagnosis and study outcomes were reviewed by at least two experts from the VTE unit on the basis of imaging studies and medical records, using a standardized data collection process to ensure consistency and accuracy.

### 2.6. Ethics and Risks

The study was conducted in accordance with the principles of the Declaration of Helsinki and Good Clinical Practice guidelines. The study protocol was approved by the Institutional Ethics Committee. All participants provided written informed consent before inclusion, and confidentiality of patient information was ensured throughout the study.

### 2.7. Statistical Analysis

Categorical variables are presented as frequencies and percentages. Continuous variables with a normal distribution are expressed as mean and standard deviation (SD), whereas non-normally distributed variables are reported as median and interquartile range (IQR). Missing data were handled using a complete-case analysis.

Associations between categorical variables were assessed using the chi-square test or Fisher’s exact test, as appropriate. Continuous variables were compared using Student’s *t* test or the Mann–Whitney U test, depending on data distribution.

Associations between systemic fibrinolysis and 30-day outcomes were evaluated using Cox proportional hazards regression models. Multivariable models were adjusted for potential confounders, including age, sex, cancer, renal insufficiency, heart rate, systolic blood pressure between 90 and 100 mmHg, RV dilatation, and combined arterial disease (including stroke, ischemic heart disease, and peripheral arterial disease). Covariates were selected based on clinical relevance and previous literature. Results are reported as hazard ratios (HRs) with 95% confidence intervals (CIs). To minimize overfitting given the limited number of outcome events, and to avoid collinearity, composite variables (such as the ESC risk classification) and derived variables (such as the shock index) were not included in the multivariable models when their individual components were already entered.

All statistical analyses were performed using SPSS software (version 20; SPSS Inc., Chicago, IL, USA) and Epidat 3.1 (Xunta de Galicia and Pan American Health Organization, Washington, DC, USA). A two-sided *p* value < 0.05 was considered statistically significant.

## 3. Results

**Figure 1:**
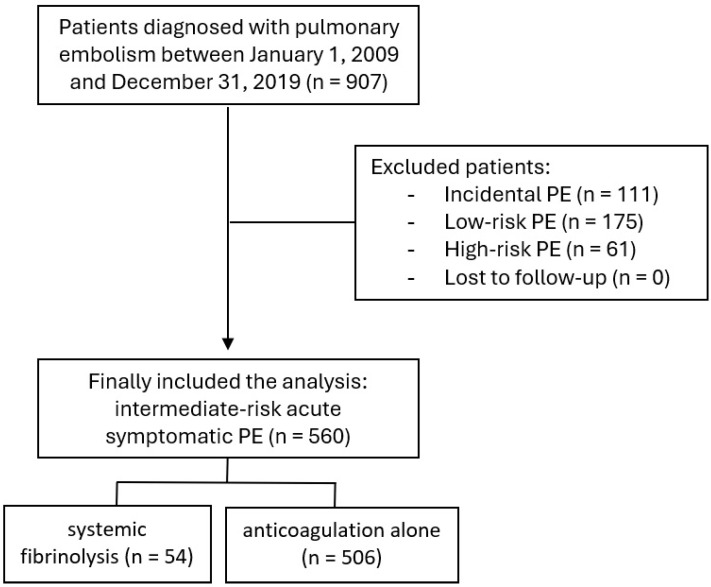
Flow-chart. PE: pulmonary embolism.

A total of 560 patients with symptomatic intermediate-risk PE were included. Of these, 54 (9.6%) received systemic fibrinolysis, whereas 506 were treated with anticoagulation alone (Figure 1). Baseline characteristics and PE presentation are summarized in Table 1.

Patients who received fibrinolysis showed signs of greater initial severity. Compared with those treated with anticoagulation alone, they had a higher median heart rate (111.5 vs. 90 beats per minute [bpm]) and a lower median systolic blood pressure (120.5 vs. 129 mmHg). Consistent with these findings, a shock index > 0.9 was more frequent in the fibrinolysis group (51.8% vs. 17.8%; *p* < 0.001). Biomarkers of myocardial injury, including NT-proBNP and high-sensitivity troponin I, were more frequently elevated in the fibrinolysis group. RV dysfunction on echocardiography (96.1% vs. 38.9%; *p* < 0.001), electrocardiographic abnormalities (73.6% vs. 44.6%; *p* < 0.001), and central PE location (75.9% vs. 34.2%; *p* < 0.001) were also more common among patients who underwent fibrinolysis.

### 3.1. Primary Outcome

At 30 days, 1 patient (1.9%) in the fibrinolysis group and 17 patients (3.4%) in the anticoagulation-only group had died (*p* = 1) (Table 2). In univariable analysis, fibrinolysis was not associated with lower 30-day all-cause mortality (HR 0.5; 95% CI 0.1–4.1). After adjustment for age, sex, cancer, renal insufficiency, heart rate, systolic blood pressure, RV dilatation, and combined arterial disease, this association remained non-significant (adjusted HR 1.5; 95% CI 0.1–17.9) (Table 3).

Continuous variables associated with 30-day mortality in univariable analyses were further evaluated using receiver operating characteristic (ROC) curve analysis to identify optimal cut-off values for risk stratification. Age > 80 years, hemoglobin < 12 g/dL, and heart rate ≥ 90 bpm were subsequently included in multivariable Cox regression models together with cancer and renal insufficiency (Table 4). In this model, age > 80 years, hemoglobin < 12 g/dL, heart rate ≥ 90 bpm, and cancer remained associated with higher 30-day mortality, whereas renal insufficiency did not.

### 3.2. Early Major Bleeding

Early major bleeding occurred in 6 patients (11.1%) in the fibrinolysis group and in 48 patients (9.5%) in the anticoagulation-only group (*p* = 0.680). Fibrinolysis was associated with a higher estimated risk of early major bleeding, although this association did not reach statistical significance in either crude or adjusted analyses (crude HR 1.96; 95% CI 0.7–5.7; adjusted HR 2.6; 95% CI 0.8–8.3) (Table 3).

Early intracranial hemorrhage occurred in 3.7% (*n* = 2) and 0.6% (*n* = 3) of patients, respectively (*p* = 0.076). The crude HR for intracranial hemorrhage within 30 days was 3.2 (95% CI 0.3–30.8) and increased to 5.2 (95% CI 0.4–62.7) after multivariable adjustment.

### 3.3. Risk Factors for Early Major Bleeding

In univariable analysis, older age was associated with a higher risk of early major bleeding. For each additional year of age, the risk increased by 4.2% (HR 1.022; 95% CI 1.003–1.042). No other variables were significantly associated with bleeding. After adjustment for potential confounders, no variable remained independently associated with bleeding within the first 30 days (Table 4).

### 3.4. Risk of 30-Day Recurrent VTE

No recurrent VTE events were observed in either group during the 30-day follow-up.

## 4. Discussion

In this prospective real-world cohort of patients with symptomatic intermediate-risk PE, the use of systemic fibrinolysis was not associated with a reduction in short-term mortality compared with anticoagulation alone. Patients selected for fibrinolysis presented with indicators of greater clinical severity; however, mortality remained low and comparable between groups. In addition, fibrinolysis was associated with a numerically higher incidence of bleeding complications. These findings should be interpreted in the context of real-world clinical practice, where treatment decisions are influenced by patient characteristics and physician judgment and may differ from the standardized conditions of randomized clinical trials. These findings should therefore be interpreted as hypothesis-generating rather than definitive evidence of treatment effect.

Patients treated with fibrinolysis exhibited a higher-risk clinical profile at presentation, suggesting that physicians preferentially selected patients with more severe hemodynamic or imaging findings for reperfusion therapy. In addition to a higher prevalence of central pulmonary embolism and tachycardia, these patients more frequently had an elevated shock index, a simple marker of hemodynamic compromise that has been associated with worse outcomes in acute pulmonary embolism and may have influenced therapeutic decision-making in routine clinical practice [21]. Despite this apparent imbalance toward greater baseline severity, short-term mortality was similar between groups. Although this observation could be interpreted as suggesting that fibrinolysis mitigated the higher baseline risk in these patients, this hypothesis cannot be confirmed due to the observational design of the study and the potential for residual confounding. Importantly, these real-world data provide insight into how fibrinolysis is applied in clinical practice, complementing evidence from randomized trials by capturing patient selection and decision-making processes that are not fully represented in controlled study settings.

Our findings are consistent with previous randomized clinical trials evaluating fibrinolysis in intermediate-risk PE [13,14]. In particular, the PEITHO trial [10] demonstrated that systemic fibrinolysis reduced the incidence of hemodynamic decompensation but did not translate into a reduction in short-term mortality. Importantly, thrombolytic therapy significantly increased the risk of major bleeding and intracranial hemorrhage. Similar conclusions have been reported in subsequent meta-analyses, which consistently show that while fibrinolysis may reduce clinical deterioration, this potential benefit is offset by an increased risk of serious bleeding complications [15]. In recent years, increasing attention has been directed toward alternative treatment strategies, such as catheter-directed thrombolysis and mechanical thrombectomy, as well as reduced-dose systemic fibrinolysis, with the aim of minimizing bleeding complications while preserving efficacy. In this context, the recently published HI-PEITHO trial showed that, in selected patients with intermediate-risk PE, ultrasound-facilitated catheter-directed fibrinolysis plus anticoagulation reduced the 7-day composite outcome of PE-related death, cardiorespiratory decompensation or collapse, or recurrent PE compared with anticoagulation alone, an effect mainly driven by fewer episodes of clinical deterioration, without an apparent increase in major bleeding or intracranial hemorrhage [20]. However, these findings refer to a catheter-directed reperfusion strategy in a relatively selected population and should not be directly extrapolated to systemic fibrinolysis. These approaches are being actively investigated and may expand the therapeutic options for selected patients with intermediate-risk PE [17,18,19].

Bleeding events remain a major concern when considering systemic fibrinolysis in patients with intermediate-risk PE. In our study, fibrinolytic therapy was associated with a higher hazard ratio for both major bleeding and intracranial hemorrhage, although these differences did not reach statistical significance after adjustment for potential confounders. These findings are broadly consistent with previous studies and highlight the importance of careful patient selection when considering reperfusion strategies [13,14,15]. Notably, increasing age was associated with a higher risk of early bleeding in the univariable analysis, a finding that has been consistently reported in studies of thrombolytic therapy [24,25]. In our cohort, patients receiving fibrinolysis were significantly younger than those treated with anticoagulation alone, likely reflecting treatment selection in routine clinical practice, where physicians may preferentially administer fibrinolysis to patients perceived to have a lower bleeding risk.

Another relevant finding of our study is the identification of several predictors of short-term mortality among patients with intermediate-risk PE. Age greater than 80 years, hemoglobin levels below 12 g/dL, and heart rate ≥ 90 bpm were independently associated with increased 30-day mortality. These findings may help refine risk stratification within the heterogeneous group of patients classified as intermediate-risk PE and may contribute to identifying patients at higher risk of adverse outcomes.

From a clinical perspective, our findings are aligned with the current guideline-recommended strategy for the management of intermediate-risk PE [3,16]. In routine clinical practice, anticoagulation alone appears to provide adequate short-term protection against mortality and recurrent thromboembolic events in most patients, while avoiding the bleeding risk associated with systemic fibrinolysis. The results of our study also illustrate how clinicians tend to reserve fibrinolytic therapy for patients with more severe clinical or imaging features, particularly younger individuals perceived to have a lower bleeding risk. These observations highlight the importance of individualized treatment decisions and careful risk–benefit assessment when considering reperfusion strategies. Our findings add to the existing evidence by providing real-world data that may help bridge the gap between clinical trial results and everyday clinical decision-making. Importantly, the role of fibrinolysis in intermediate-high risk PE remains an area of active investigation and has not been abandoned. Ongoing randomized trials such as PEITHO-3 are evaluating reduced-dose alteplase in this population in an effort to improve the safety profile of systemic fibrinolysis and optimize the balance between efficacy and bleeding risk and are expected to provide important evidence in the near future [19].

Several limitations should be acknowledged. First, although data were collected prospectively, the observational design introduces the possibility of residual confounding and treatment selection bias, particularly given that fibrinolysis was administered at the discretion of the treating physician. Second, baseline differences between treatment groups reflect confounding by indication, as patients receiving fibrinolysis had more severe clinical and hemodynamic features. Although multivariable adjustment was performed, residual confounding cannot be excluded. Furthermore, the number of outcome events limited the number of variables that could be included in the models, which may have influenced the adjusted estimates. Third, the timing and specific criteria for systemic fibrinolysis were not standardized and were left to the discretion of the treating physician, which may have introduced variability in treatment decisions and reflects the heterogeneity inherent to real-world clinical practice. Finally, the study was conducted at a single tertiary center, which may limit the generalizability of the findings to other healthcare settings.

Despite these limitations, the study also has several strengths. The prospective inclusion of consecutive patients through a dedicated institutional VTE registry minimizes selection bias and ensures systematic data collection. In addition, the study reflects real-world clinical practice over a prolonged study period and includes detailed information on clinical presentation, imaging findings, and outcomes.

In conclusion, in this real-world cohort of patients with symptomatic intermediate-risk PE, systemic fibrinolysis was not associated with a reduction in 30-day mortality compared with anticoagulation alone and was associated with a numerically higher risk of bleeding complications. These findings are in line with current guideline recommendations, although no causal relationship can be established due to the observational nature of the study.

## Figures and Tables

**Table 1:** Baseline characteristics, clinical, laboratory, and imaging characteristics of the episode and prognostic scores of patients with acute symptomatic intermediate-risk PE.

Variable	Systemic Fibrinolysis (*n* = 54)	Anticoagulation Alone (*n* = 506)	*p* Value
Baseline characteristics			
Median age, years (IQR)	58 (43–70)	73 (58–82)	<0.001
Male sex, *n* (%)	32 (59.3)	223 (44.1)	0.033
BMI (Kg/m^2^), (median, IQR)	26.9 (23.9–32.7)	28 (25.1–31.7)	0.439
Diagnosed in the ED	52 (96.3)	450 (89.3)	0.103
Antiplatelet therapy, *n* (%)	6 (11.1)	112 (22.4)	0.055
Coronary artery disease, *n* (%)	3 (5.6)	45 (8.9)	0.608
Stroke, *n* (%)	0	34 (6.7)	0.064
Peripheral artery disease, *n* (%)	2 (3.7)	23 (4.6)	1
Hypertension, *n* (%)	21 (38.9)	293 (57.8)	0.008
Diabetes, *n* (%)	13 (24.1)	80 (15.8)	0.123
Chronic anticoagulation, *n* (%)	0 (0)	6 (1.9)	1
Major bleeding in the previous month, *n* (%)	0	21 (4.1)	0.248
Chronic heart failure, *n* (%)	1 (1.9)	53 (10.4)	0.041
Atrial fibrillation, *n* (%)	1 (1.9)	21 (4.1)	0.495
Chronic pulmonary disease, *n* (%)	4 (7.4)	94 (18.6)	0.040
Chronic kidney disease, *n* (%)	2 (3.7)	78 (15.4)	0.019
Active cancer, *n* (%)	5 (9.3)	150 (29.7)	0.001
Provoked VTE, *n* (%)	23 (42.6)	273 (53.9)	0.112
PE presentation			
Length of stay, days (median, IQR)	8 (6–10)	8 (6–12)	0.349
Central PE ^&^, *n* (%)	41 (75.9)	173 (34.2)	<0.001
Heart rate (median, IQR)	111.5 (101–125)	90 (79–110)	<0.001
Blood pressure, mmHg (median, IQR)	120.5 (103–140)	129 (115–144)	0.025
Oxygen saturation_,_ % (median, IQR)	93 (89–96)	93 (90–95)	0.673
Syncope, % (*n*)	11 (20.4)	72 (14.2)	0.227
Concomitant DVT, *n* (%)	15/26 (57.7)	161/270 (59.6)	0.843
Laboratory tests			
Hemoglobin, g/dL (median, IQR)	14.6 (12.8–15.6)	13.3 (12–14.5)	0.001
Platelets, /mm^3^ (median, IQR)	186 (147–224)	201 (161–251)	0.055
Elevated hs-cTnI *, *n* (%)	38/44 (86.4)	150/290 (51.7)	<0.001
D-dimer, ng/mL (median, IQR)	1252 (1100–2967)	1875 (1028–3490)	0.651
NT-proBNP > 600 ng/L	27/42 (64.3)	132/287 (45.9)	0.027
Echocardiogram			
RV hypokinesis, *n* (%)	49/51 (96.1)	155/399 (38.9)	<0.001
Computed tomography			
IVC contrast reflux, *n* (%)	33/45 (73.3)	132/411 (32.1)	<0.001
Simplified PESI score			
sPESI ≥ 1, *n* (%)	38 (70.4)	425 (83.9)	0.012
ESC score			
Intermediate-low risk, *n* (%)	15 (27.8)	400 (79.1)	<0.001
Intermediate-high risk, *n* (%)	39 (72.2)	106 (21)	
Shock Index			
Shock Index > 0.9 ^	28 (51.8)	89 (17.8)	<0.001

* hs-cTnI (elevated high-sensitivity cardiac Troponin): concentrations of greater than 15.6 pg/mL in women and 34.2 pg/mL in men were considered as positive according to the laboratory normal range. ^&^: Pulmonary trunk or main pulmonary arteries involvement. ^: Heart rate was missing in 6 patients. BMI: body mass index; ED: emergency department; ESC: European Society of Cardiology; DVT: Deep vein thrombosis; IQR: interquartile range; IVC: inferior vena cava; PE: pulmonary embolism; sPESI: simplified Pulmonary Embolism Severity Index; RV: right ventricle; VTE: venous thromboembolism.

**Table 2:** Treatment in patients with hemodynamically stable acute symptomatic intermediate-risk pulmonary embolism.

Variable	Systemic Fibrinolysis (*n* = 54)	Anticoagulation Alone (*n* = 506)	*p* Value
Acute phase treatment (0–21 days)			
Low molecular weight heparin, % (*n*)	49 (90.7%)	480 (94.9%)	0.208
Unfractionated heparin, % (*n*)	33 (77%)	44 (8.7%)	<0.001
DOACs, % (*n*) (rivaroxaban or apixaban)	7 (12.9%)	48 (9.5)	0.414
Treatment phase (first 3–6 months)			
Vitamin-K antagonists, % (*n*)	43 (79.6%)	286 (56.5%)	0.001
DOACs, % (*n*)	18 (33.3%)	128 (25.3%)	0.201
Other treatments during acute phase			
Catheter-directed thrombectomy, % (*n*)	0 (0%)	8 (1.6%)	0.352
Surgical embolectomy, % (*n*)	0 (0%)	5 (0.9%)	0.463

DOACs: direct oral anticoagulants.

**Table 3:** Association between systemic fibrinolysis and 30-day outcomes: univariable and multivariable Cox proportional hazards regression analysis in patients with intermediate-risk pulmonary embolism.

Systemic Fibrinolysis	Univariable Analysis (Crude HR)	Multivariable Analysis (Adjusted HR)
	Hazard Ratio (CI 95%)	
30-day mortality	0.5 (0.1–4.1)	1.5 (0.1–17.9) *
30-day major bleeding	1.9 (0.7–5.7)	2.6 (0.8–8.3) ^%^
30-day intracranial hemorrhage	3.2 (0.3–30.8)	5.2 (0.4–62.7) ^%^

* Adjusted for: age, sex, presence of cancer, renal insufficiency, heart rate, systolic blood pressure between 90–100 mmHg, right ventricular dilation, and combined arterial disease (including stroke, ischemic heart disease, and peripheral arterial disease). ^%^ Adjusted for: age, sex, active cancer, renal insufficiency, and arterial disease. HR: Hazard Ratio. CI: confidence Interval.

**Table 4:** Univariable and multivariable Cox proportional hazards analysis of predictors of 30-day mortality and major bleeding in patients with intermediate-risk pulmonary embolism.

	HR	95% CI	HR	95% CI
	Univariable		Multivariable	
30-day mortality				
Active cancer	3.3	1.31–8.4	2.9	1.1–7.6
Chronic kidney disease	3.3	1.31–8.3	2.4	0.9–6.1
Age > 80	5.2	1.93–13.7	5.5	2.0–15.2
Hemoglobin < 12 g/dL	5.1	1.99–13.2	4.0	1.5–10.7
Heart rate ≥ 90 bpm	3.6	1.03–12.4	5.2	1.5–18.3
30-day major bleeding				
Age	1.02	1.0–1.04	1.0	0.9–1.0
Hemoglobin	1.1	0.9–1.3	1.1	0.9–1.3
Syncope	0.6	0.3–1.3	0.6	0.3–1.3
Central PE location	1.3	0.7–2.3	1.3	0.5–1.6
Active cancer	0.9	0.5–1.7	0.8	0.4–1.6
Chronic kidney disease	1.7	0.9–2.9	1.3	0.7–2.4
Major bleeding in the previous month	0.9	0.2–4.1	1.3	0.3–5.6

CI: confidence Interval; HR: Hazard ratio; PE: pulmonary embolism.

## Data Availability

Data are not publicly available due to ethical and institutional restrictions related to patient confidentiality. Anonymized data may be made available from the corresponding author upon reasonable request.
